# The role of health system context in the design and implementation of performance-based financing: evidence from Cote d’Ivoire

**DOI:** 10.1136/bmjgh-2020-002934

**Published:** 2020-09-30

**Authors:** Denizhan Duran, Sebastian Bauhoff, Peter Berman, Tania Gaudet, Clovis Konan, Emre Ozaltin, Margaret E Kruk

**Affiliations:** 1 Health, Nutrition and Population, World Bank Group, Washington, District of Columbia, USA; 2 Department of Global Health and Population, Harvard T.H. Chan School of Public Health, Boston, Massachusetts, USA; 3 The University of British Columbia School of Population and Public Health, Vancouver, British Columbia, Canada; 4 Ministry of Health of Cote d'Ivoire, Abidjan, Côte d'Ivoire; 5 Human Development, World Bank Group, Maputo, Mozambique

**Keywords:** health policy, health systems evaluation, health systems

## Abstract

Low quality of care is a significant problem for health systems in low-income and middle-income countries (LMICs). Policymakers are increasingly interested in using performance-based financing (PBF), a system-wide provider payment reform, conditioned on both quantity and quality of performance, to improve quality of care. The health system context influences both the design and the implementation of these programmes and thus their effectiveness. This study analyses how context has influenced the design and implementation of PBF in improving the quality of primary care in one particular setting, Cote d’Ivoire, a lower-middle income country with some of the poorest health outcomes in the world. Based on literature, an analytical framework was developed identifying five pathways through which financial incentives can influence the quality of primary care: earmarking, conditioning, provider behaviour, community involvement and management. Guided by this framework, semistructured interviews were conducted with policymakers and providers to diagnose the context and to assess the links between financing and quality of care at the primary care level. PBF in Cote d’Ivoire was found to have increased data availability and quality, facility-wide and disease-specific inputs, provider motivation and management practices in contracted facilities, but had limited success in improving process and outcome measures of quality, as well as community involvement and the provision of non-incentivised services. These limitations were attributable to a centralised health system structure constraining the decision space of health providers; financing and governance challenges across the health sector; and shortcomings with regard to the design of the PBF quality checklist and incentive structures in Cote d’Ivoire. In order to improve the quality of primary care, health sector reforms such as PBF should incorporate the organisational and service delivery context more broadly into their design and implementation, as is the case in other countries.

**Key questions**

**What is already known?**

- Performance-based financing (PBF) has been implemented in over 50 lower-income and middle-income countries, where it has succeeded in improving service utilisation and structural and process measures of quality of care.

**What are the new findings?**

- In contracted facilities in Cote d’Ivoire, PBF resulted in improvements in data availability and quality, levels of facility-wide and disease-specific inputs, and provider motivation and management practices.
- It was less successful in improving process and outcome measures of quality.
- These limits were attributable to an overly centralised health system constraining the decision space of health providers; financing and governance issues across the health sector; and the design of the quality checklist and incentive structures.

**What do the findings imply?**

- Accounting for organisational and service delivery context in the design and implementation of health sector reforms, as well as a consideration of political economy factors, can improve such reforms’ effectiveness in improving quality of primary care.

## Introduction

Low quality of care is a significant problem in many low-income and middle-income countries (LMICs): in 2016, 8.6 million excess deaths were attributable to low quality care, of which 5 million were due to poor-quality care and 3.6 million were due to non-utilisation.^1^ This is particularly a problem for primary care, which continues to be the first point of contact with the health system for many people, especially in LMIC such as Cote d’Ivoire, where over 70% of care is sought at the primary level.^2^

Interventions to improve the quality of health systems can be implemented at the facility and the system levels, involving both health system contexts and other systems impacting the health sector. Common approaches focus on organisational design, management and organisational processes, and incentives.^3^ In order to improve quality of care, policymakers have predominantly used clinic-level or provider-level interventions, with limited effectiveness.^4^ Recent research emphasises the role of health system-level factors such as financing and governance on improving quality of care.^5^

Financing is one of the most frequently used system-wide interventions,^6^ and performance-based financing (PBF) is one such intervention that has been piloted in over 50 LMICs with the aim of improving access and quality of care. PBF pays health providers or facilities and also other entities in the health system such as districts or regions, directly for the achievement of a predefined set of quality and quantity targets.^7^ Many PBF programmes have been evaluated on their effects on utilisation, and programmes are increasingly being evaluated on their impact on quality of care.^8^ These evaluations demonstrate that through collecting data, bringing flexible funding to health centres and incentivising health providers, PBF programmes can generate improvements in structural dimensions of quality of care at health facilities, and catalyse the implementation of purchasing and contracting reforms.^9^

PBF in Cote d’Ivoire was launched in 2016 as a pilot project financed by the World Bank, with the objective of jumpstarting the health system and improving service utilisation and quality after a prolonged period of instability and low levels of investment in the health sector. While the programme will go through a full impact evaluation, an analysis of administrative data points to increases in incentivised utilisation and quality indicators. (Administrative data were analysed by the lead author as part of this project.) The government is currently in the process of rolling out health insurance and intends to scale up PBF nationally by 2021.

There is a dearth of research on the mechanisms through which the health system context influences the design and implementation of quality improvement interventions such as PBF. Given the complexity of health systems, it is imperative to diagnose the context in which a system-wide intervention is being implemented.^10^ Diagnosing the baseline health system context to align implementation fidelity with environmental conditions can contribute significantly to design and implementation success.^3^

This study examines the influence of the health system context on the design and implementation of PBF in Cote d’Ivoire as it pertains to improving the quality of primary care. We developed a theory of change that includes contextual factors and mechanisms through which PBF can influence quality of care, and used this framework to study on-the-ground implementation of PBF. Specifically, we assess how the baseline health system context enabled or constrained the effectiveness of PBF in improving the quality of primary care. We find that the limitations in the Ivorian health system context led to a successful but partial improvement in quality of care, and larger system-level reforms may be needed to achieve more significant improvements in quality of primary care.

## Methods

### Setting and intervention

Cote d’Ivoire is a lower-middle income country in West Africa that in 2011 emerged from a prolonged period of political instability. Its health outcomes are among the poorest in the world, with a life expectancy of 55 and a maternal mortality rate of 645/100 000.^2^ Due to low levels of investment in the health system, 30 000 deaths in Cote d’Ivoire were attributable to poor quality of care in 2015, which is also one of the highest rates in the world.^1^ These poor outcomes can be attributable to low and fragmented levels of overall and public health spending.^2^ Only 9% of the government’s health spending is targeted towards primary care, even though over 70% of care was sought at this level in 2017.^2^ The low levels of financing are exacerbated by the lack of strategic purchasing mechanisms, and fragmented and inadequate transfers from the government to primary health facilities.^2^ Table 1 demonstrates indicators pointing out to low health system capacity in Cote d’Ivoire.

**Table 1:** Key indicators for Cote d’Ivoire^2^

	Data point	Year
***Overall indicators***		
Population	24 290 000	2017
GDP per capita, constant 2010 USD	$1626	2017
GDP growth rate	7.70%	2017
Poverty rate at $1.90/day	28.20%	2015
***Key health outcome indicators***		
Share of communicable diseases (% of overall disease burden)	63%	2017
Life expectancy at birth	55	2017
Maternal mortality rate (/100 000)	645	2017
Under 5 mortality rate (/1000)	91	2016
***Key health financing indicators***		
Per capita health expenditure (USD)	70	2016
Public (%)	25%	2016
Out of pocket (%)	48%	2016
Private (%)	12%	2016
External (%)	15%	2016
Pooled (%)	21%	2016
***Key service delivery indicators***		
Daily patients per health centre	13	2016
% of population living outside a 5 km radius from any health facility	33%	2017
Access to transport system for health centres	34%	2016
Average operational capacity at health centres	56%	2015
% of health centres complying with infection prevention standards	22%	2015
Essential medicine availability at health centres	28%	2015
Diagnostics availability at health centres	4%	2015
% of health centres on a power grid	83%	2016
Health centres with access to piped water	47%	2016
Health centres with laboratory services	16%	2016
Health centres with basic surgery offered	78%	2016

GDP, gross domestic product.

In order to improve the quality and quantity of health services at the primary care level, PBF was first piloted in 2016 in 401 primary health centres in 19 districts of Cote d’Ivoire, covering about a quarter of the entire population, with World Bank financing. The contracted health centres first receive quarterly subsidies based on the quantity of services they provided, from a package of 24 maternal, newborn and child health, outpatient visits, HIV and tuberculosis (TB) interventions. In addition, health centres are supervised quarterly by district health offices and are assessed based on a 149-item checklist focusing on disease-specific, facility-wide and management-related inputs (table 2). This checklist is used to pay health centres a quality bonus, calculated by multiplying the total quantity payment with the quality score (0%–100%) from that quarter. Once subsidies are received, facilities pool them and are mandated to spend 50% on incentives paid directly to staff, and the remaining 50% on infrastructure, rehabilitation and operating costs, with restrictions on using these funds for recurrent expenditures such as drugs and medical supplies or hiring health workers directly. Management and administration committees at the health centre level oversee the planning, budgeting and finance processes.

**Table 2:** PBF quality checklist and indicator summary

PBF category	Framework dimension	No. of indicators	Weight
General indicators	Management	13	24
Budgeted business plan	Management	4	10
Financial management	Management	3	5
Hygiene, safety, environment	Facility-wide inputs	14	27
Outpatient consultation and emergencies	Facility-wide inputs	16	20
Vaccination	Disease-specific inputs	19	30
Maternity	Disease-specific inputs	22	32
Family planning	Disease-specific inputs	8	13
Antenatal visits	Disease-specific inputs	5	14
Guardroom	Management	4	8
Malaria case management	Disease-specific inputs	4	8
ARI, diarrhoea and TB case management	Disease-specific inputs	3	6
HIV case management	Disease-specific inputs	12	22
Pharmacy	Facility-wide inputs	6	12
Availability of tracer drugs	Facility-wide inputs	16	16
**Total**		**149**	**247**
**Disease-specific inputs** (**Conditioning**)		**73**	**125**
**Facility-wide inputs** (**Earmarking**)		**52**	**75**
**Management** (**Management and institutional reform**)		**24**	**47**

ARI, acute respiratory infections; TB, tuberculosis.

### Policy question and analytical framework

This paper assesses how the Ivorian health system context has influenced the implementation and effectiveness of PBF with regard to its ability to improve the quality of primary care. It does so through first developing an analytical framework identifying mechanisms through which PBF can influence quality of primary care, based on a review of PBF studies in LMIC. It then uses an implementation research approach^11 12^ through utilizing this framework in assessing the implementation and effectiveness of PBF, primarily through qualitative interviews conducted with key stakeholders at policy and service delivery levels. In order to assess quality of care at the primary level, this study uses and adapts the Lancet Commission for High Quality Health Systems (HQSS) framework as per Macarayan *et al*,^13^ including three components:

- Competent systems: safety, prevention and detection, continuity and integration, population health management and timely action.
- Evidence-based care: technical quality indices regarding systematic assessment, correct diagnosis, appropriate treatment and counselling for each visit.
- Positive user experience: patient focus and clear communication.

**Figure 1:**
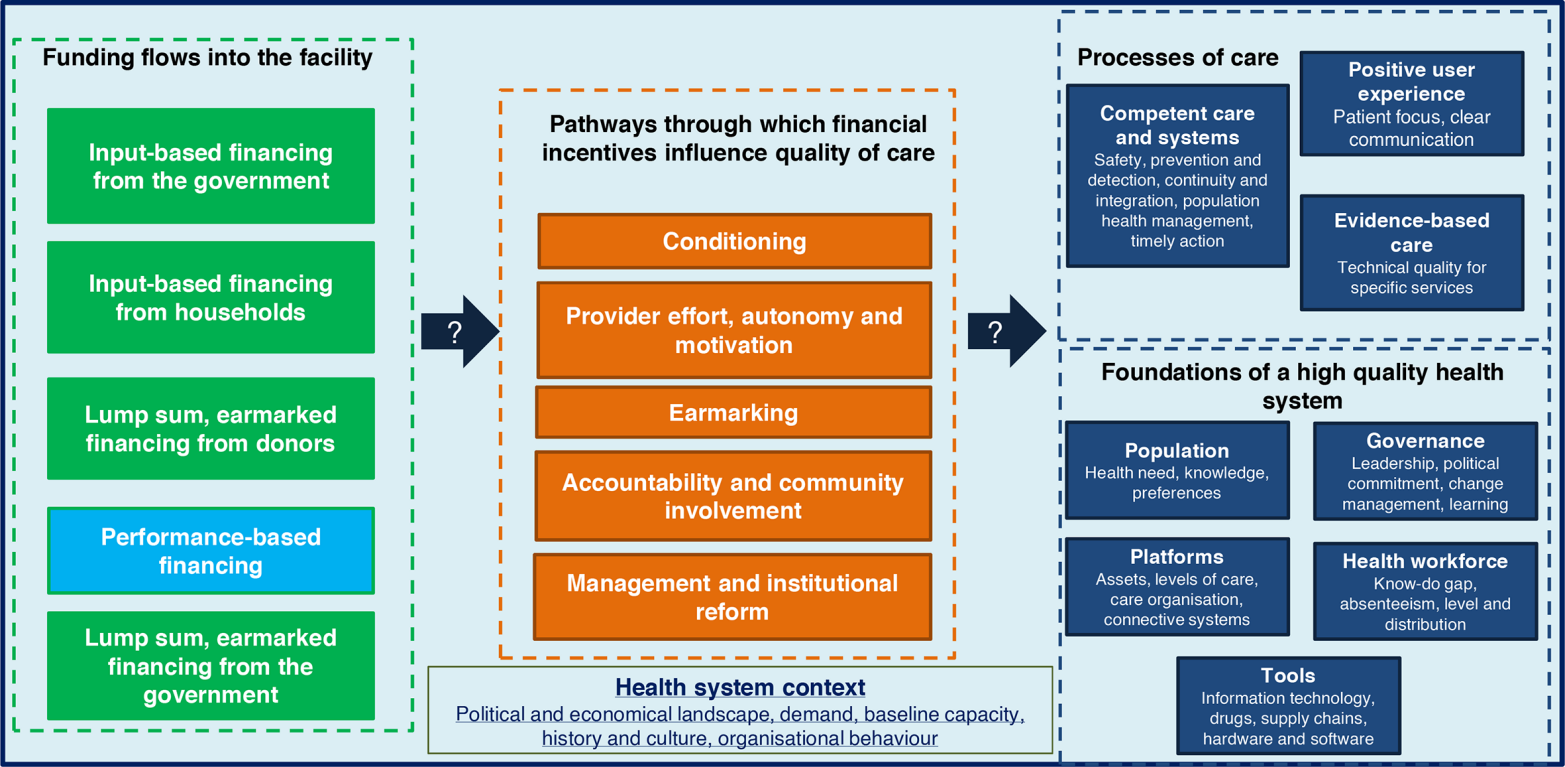
Analytical framework.

The analytical framework (figure 1) consists of five pathways through which PBF can influence quality of care: conditioning, provider behaviour, earmarking, accountability and community involvement, and management and institutional reform^6 14 15^ **Conditioning** refers to the transfer of funds to health facilities given the achievement of a predefined set of requirements; **provider behaviour** pertains to how the satisfaction, motivation and autonomy of health workers would respond to financial incentives and improve provider effort, absenteeism and their know/do gap; **earmarking** and the flexibility associated with using the funds once they come in to the facility has the potential to give providers more autonomy to be responsive to issues at the service delivery level; improving **accountability and community involvement** can take place through involving the community in quality of care improvement either as a direct condition for providing payment or through supplemental activities related to PBF projects; and finally, **management and institutional reform** pertains to the increase in the ability of providers to proactively plan and execute funds due to the improvement of data availability, supervision visits and flexibility of funds associated with financial incentives.

### Qualitative data collection and analysis

Semistructured interviews were conducted with 82 key informants in 25 sessions at central, district and facility levels to gain an understanding of the context and to document the pathways through which PBF has influenced quality of care. (Stakeholders at the central and district levels included officials from Ministry of Health directorates focusing on health financing, service delivery and quality of care, as well as development partners included in PBF implementation. Stakeholders at the facility level included facility in-charges, facility management committees and health workers (primarily nurses and midwives).) Districts and facilities were selected based on PBF implementation, PBF performance (level and improvement) and facility type (urban vs rural). Seven districts were visited, five of which were PBF districts and two were non-PBF districts; in each of the districts, at least one health centre was visited. Interview guides in French were developed based on the analytical framework, and verbal and written consent was acquired by interviewees; every interview was recorded. While the framework was used as a reference point, the interviews also included open-ended questions focusing on the overall perspectives of interviewees on the Ivorian health system, particularly with regard to financing and quality of care; these insights are included in the ‘Policy context’ section. Confidentiality was assured as identifying characteristics were not recorded and individual quotes or emerging themes were not associated with any individual. The data collected from the interviews were then analysed using the framework analysis method consisting of five steps: familiarisation, identifying a thematic framework, indexing, charting, and mapping and interpretation.^16^ During the interviews, field notes were taken, which were organised in tables around the different components of the framework, with results organised around emerging themes.

## Results

**Figure 2:**
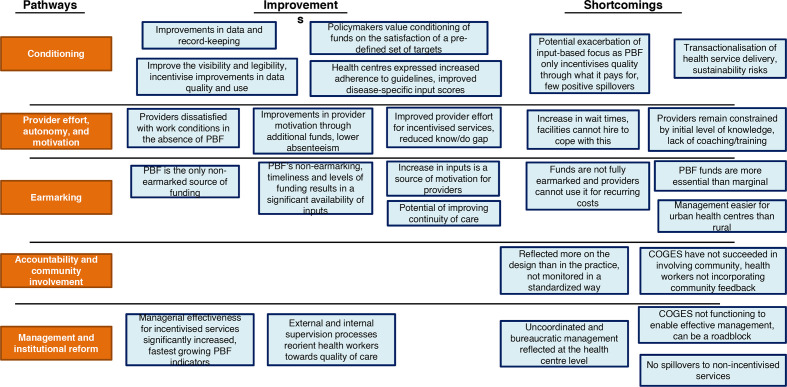
Qualitative findings by each of the five mechanisms. PBF, performance-based financing. COGES, comité de gestion (management committee at health centres)

The results of this study are presented in two parts: first, the results of the diagnosis of the Ivorian policy context; and second, the implementation experience through the five pathways outlined in the theory of change. Results across pathways are demonstrated in figure 2.

### Policy context

Analysis of interview data and national policy documents points to the lack of a common and comprehensive vision for quality of primary care from the central level to the provider level. Stakeholders’ definitions of quality of care focused on structural aspects of quality of care and excluded other dimensions: for example, one of the senior health system directors at the Ministry of Health indicated that the most significant problem they face is “the lack of resources to disseminate guidelines to health centers on quality of care,” whereas others highlighted scarcity of human and physical resources. Other significant dimensions of quality of care based on the HQSS framework such as safety, prevention and detection, continuity and integration, population-health management, timely action or those that pertain to provider competence such as evidence-based care, patient focus and clear communication, were left out of the definition of almost every stakeholder across all levels of the health sector. At the health centre level, the definition for quality of care focused on visible aspects, such as ‘welcoming the patients’ or ensuring physical inputs are present, which are necessary but not sufficient for ensuring high quality of care. At the central level, this over-reliance on quick and shorter-term fixes was also evident in certain policy decisions that were taken recently, such as the upgrade of rural health centres to urban health centres, or of urban health centres to first level hospitals, without a corresponding increase in the capacity of physical or human resources.

**Figure 3:**
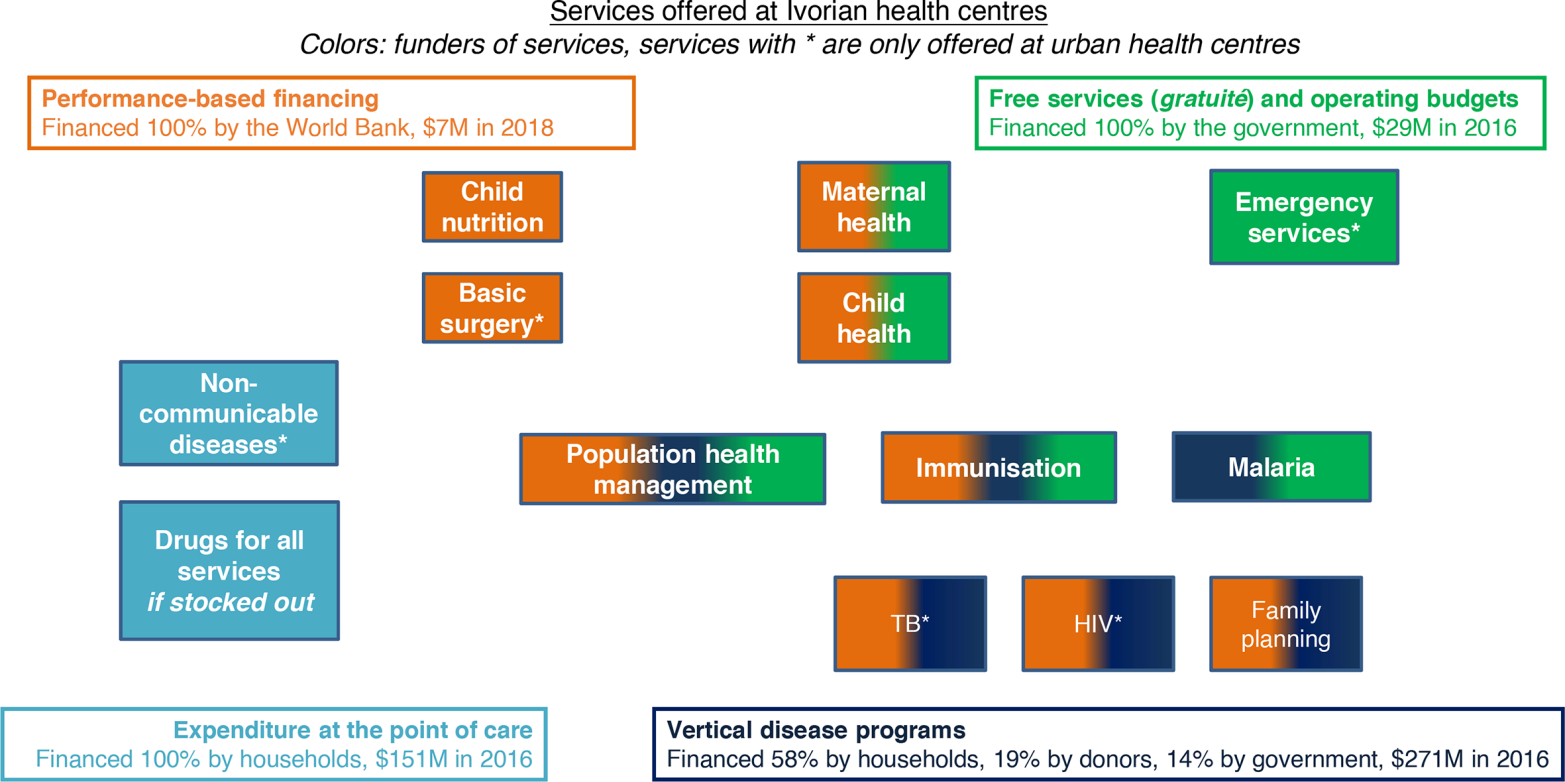
Services offered at Ivorian health centres and payers. TB, tuberculosis.

Similar to challenges in governance, financing flows are not aligned to send coherent signals to primary health facilities. As a result, it is not possible to purchase high-quality healthcare processes or outcomes across the revenue raising, pooling and purchasing functions. In terms of revenue raising, interviewees reported that the government does not spend sufficiently to enable high-quality care, resulting in interruptions in service delivery as well as high out-of-pocket spending. In the absence of a unified risk pool, health centres face significant fragmentation in financing flows for individual services: as can be seen in figure 3, service delivery at health centres remains fragmented, which results in mixed signals sent to health facilities with regard to prioritisation of services and quality improvement. Further, services are purchased from primary care facilities based on historical allocation and not need, utilisation, performance or equity considerations. Providers highlighted significant restrictions on spending funds that come into the health facilities: for example, health centres only retain about 15% of user fees, and overall have very low of flexible financing to deliver interventions. As a health worker from an urban health centre without PBF indicated, “The government funds, at best, enable us to find palliative solutions to a disease that has to be cured. We do not have the funds that we need to deliver high quality care, and the current arrangements do not permit us to use the money for what we want, even if we get the money.” Given the lack of other funding sources in health facilities, PBF was associated with a significant financing infusion at the health centres where it was implemented, making up to 60% of the facility budget and presenting sustainability challenges. (Based on an analysis of PBF financial data.)

### Implementing PBF: intent and reality

On its introduction in Cote d’Ivoire, PBF comprehensively defined the components of a high-quality health system for the first time for key stakeholders. However, PBF’s definition of quality of primary care is by definition limited as it is incentivised through a checklist, which is neither comprehensive nor exhaustive: a review of the Ivorian PBF checklist demonstrates its similarity to other PBF checklists,^8 17^ with a reliance on structural indicators and significantly fewer process or outcome-related indicators. Less than 10% of the 149 indicators focus on processes of care, with the rest focusing primarily on disease-specific or facility-wide inputs. Further, these input indicators do not capture many dimensions of a high-quality health system as per the HQSS framework, such as timely action, patient focus, clear communication, adverse events, facility-associated infections or improper medical practices. Prevention and detection, as well as continuity and integration, are also not directly monitored in the quality checklist, even as some indicators indirectly measure them.

According to policymakers, **conditioning** was the primary pathway through which PBF could improve quality of care, specifically through improving the coherence of financing signals to health facilities, incentivising the government to focus more on quality of care and improving the availability of data in the primary healthcare system. Through conditioning bonuses based on a quality checklist, PBF has improved data availability, with high reporting rates and publicly available data across the tracked quantity and quality indicators. In this regard, PBF’s ‘catalytic’ potential was shared by policymakers, and the way that PBF made the health sector ‘visible’ through the availability of data was a sentiment shared by policymakers across all levels. Given the pre-existing input-focused approach to defining quality of care and the fact that PBF only incentivises quality through what it pays for, providers demonstrated a tendency to think about quality of care only as defined and incentivised by PBF: almost every stakeholder interviewed defined quality exclusively within the PBF checklist. Further, even though resources have not explicitly been taken away from non-incentivised services, the newly available resources were rarely made available to improve the quality and quantity of non-incentivised services. For example, a PBF health centre purchased a blood pressure monitoring device, but indicated that they only use it for antenatal care visits and not for non-communicable disease (NCD) screening for other potential at-risk populations, as these were not incentivised within the PBF package, although it should be noted that NCD provision has been low in Cote d’Ivoire prior to PBF implementation as well, demonstrating the limits imposed by conditioning incentives on a subset of services. Similar patterns were seen in the way outreach and community visits were conducted, focusing only on maternal and child health, as opposed to incorporating other conditions. Another concern expressed by health workers was with regard to the sustainability of conditioning, noting that “if PBF disappears, quality scores will drop faster than the decline in funds,” which was also seen in facility budgets as PBF funds constituted a large portion of facility budgets, an issue which potentially can be rectified with the planned transfer of PBF funds into the government’s own budget as well as improving the overall level of financing in the health sector.

In a context of poor provider motivation due to poor working conditions and low compensation, improvements in **provider effort** in PBF facilities were observed through adherence to clinical guidelines and a reduction of the know/do gap, longer work hours and reduced absenteeism, and these were attributable to both financial incentives and the associated autonomy with these incentives. At PBF facilities, health workers receive a share of the subsidies based on their absenteeism, experience, individual performance and position, and health workers indicated that this has increased their salary by more than 30%. The PBF programme validates adherence to clinical guidelines through reviewing patient registries for a subset of services (eg, whether a partogram was filled during a delivery), and as such, it is not possible to document quantitative improvements in provider effort. Interviews with health workers demonstrated increased working hours, attributable to increased demand resulting from better structural quality at health centres as well as provider outreach to communities to improve service utilisation, as well as decreased absenteeism as it is directly being measured. Certain health workers also highlighted that PBF made them feel empowered, as they were given clear instructions about what performance meant and received more tools and guidance to be able to deliver on their tasks. Similar to conditioning, however, health workers explicitly mentioned that they prioritised service delivery and reporting for the services that are under PBF. One final limitation, outside of the purview of PBF, was that health workers remained constrained by their initial level of knowledge, as in-service training curriculum was not updated based on health worker performance; as such, even though performance improved, it only improved up to the initial knowledge capacity of health workers.

At the facility level, the most significant benefit of PBF highlighted by health workers was an increase in non-**earmarked** financing resulting in improved inputs and innovation, even though there were certain restrictions. In a context where most funds are earmarked, PBF is the most significant source of flexible funding at the health centre level. Providers indicated their ability to use these funds in a more agile way, using them for improving the availability of medical equipment, water, electricity as well as improved stock and waste management. The improvements in resources were also associated with higher provider motivation, especially compared with health centres without PBF which were found to lack inputs to deliver the services they were mandated to provide. Further, PBF funds gave providers the opportunity to innovate, even though this innovation was focused on incentivised services, such as more outreach to build demand for maternal health services, or investing in the infrastructure of the health centre to facilitate triage treat more patients concurrently. Yet, PBF funds are not fully flexible due to Ministry of Health restrictions: they are not pooled with the other funds at the health centre level and they cannot be used to finance the recurrent transaction budgets at health centres, which prohibits providers from using them to purchase drugs or fuel. This was the most significant frustration expressed by providers at the health centre level, as they faced regular stock-outs with essential medications and did not have fuel for their ambulances.

PBF in Cote d’Ivoire was also designed with the intention of improving **accountability and community involvement**, as well as strengthening **management** at the facility level. Even though PBF sought to improve accountability through mandating providers to collect patient feedback, these checklists were not standardised, and the PBF programme only rewarded the availability of a mechanism to collect patient feedback, as opposed to whether the feedback was addressed. As such, even though many providers and policymakers listed patient satisfaction as a key dimension of quality of care, almost none of them were able to indicate specific actions they undertook to improve patient satisfaction. PBF has also succeeded in establishing the institutions at the facility level to potentially do so: every PBF facility has a management committee consisting of community members and health facility staff, deciding how the PBF funds are allocated through budgeted business plans, and overseeing internal and external supervision processes through which explicit steps to improve quality of care can be determined at the facility level. Health providers, facility in-charges and district offices all welcomed these processes as strong levers in quality improvement, especially considering the absence of management processes in facilities without PBF. However, in certain facilities, management committees were not working well, as they were not being incentivised to meet. While management processes were intended to include all services, interviews and a review of plans demonstrated that non-PBF services at the facility were not prioritised from a quality or quantity perspective. This limitation was due to the fact that the organisational structure change in the health centres was not matched with autonomy at the central level: health facilities cannot hire or fire health workers, and do not have authority over planning or allocating the non-PBF part of their budget, an issue also seen in other settings where autonomy at the facility level is lacking.

## Discussion

In a relatively short period of 2 years, PBF in Cote d’Ivoire has improved data availability, availability of medical and non-medical equipment, provider effort and motivation, and management processes for incentivised services. In this regard, PBF has served as an entry point to the reorientation of the Ivorian health system towards an integrated vision for quality of primary care. However, PBF’s impact has been less pronounced on the dimensions of quality of care that were not directly incentivised by PBF. These shortcomings can be attributed to the Ivorian health system context constraining the PBF programme’s success, as well as with shortcomings in the Ivorian PBF programme design exacerbating health system constraints.

The Ivorian health system’s emphasis on input-oriented financing over performance, fragmented governance structure, lack of autonomy for decentralised entities and low level of flexible health system financing have constrained the design and implementation of PBF. First, an overreliance on physical inputs as measures of performance, such as infrastructure and equipment, has guided the way that PBF funds have been spent and indicators have been designed. Second, the fragmented governance structure in which there is no single directorate for quality of primary care has led to an incoherence of signals being sent to the service delivery level, enabling PBF indicators to play an outsized role in defining quality. Third, the lack of fiscal autonomy for decentralised entities has limited the responsiveness of health centres to any potential challenges with quality of care, such as purchasing drugs and fuel, or recruiting health workers to respond to increases in demand.

The design of PBF in Cote d’Ivoire also faced certain shortcomings. First, as it has defined quality only through a checklist which for the most part has included physical inputs, it further reinforced a definition for quality of care which is more focused on inputs for a specific set of services. Given its role as the premier source of flexible funding at the facility level, PBF has significant leverage to result in facility-wide improvements of quality of care; yet, due to its design in Cote d’Ivoire, its impact on improving processes or outcomes of care has been muted. Having been implemented in a health system without a coherent definition for quality of care, it mirrored a focus on inputs as well as a focus on vertical programmes. As such, while PBF was meant to improve quality for the entirety of the health system across all levels, it ended up doing so predominantly for incentivised indicators and services.

Even as few studies explicitly link country context to PBF implementation and its impact on quality of care, these results are in line with other evaluations: across a wide range of countries, PBF’s impact on quality was found to benefit structural dimensions of quality more than process and outcome measures.^18^ A recent study from Mali using a very similar approach^19^ also identified the significant role of baseline implementation context, specifically in terms of the role and capacity of decentralised entities prior to implementation. Studies from Benin, Democratic Republic of the Congo, Rwanda and Zambia demonstrate that the conditionality aspect of PBF is associated with improved reporting and improved provider motivation, improved provider effort for incentivised services, with limited to no positive impact for non-incentivised services.^20–23^ Certain studies assessing the role of flexibility of funds from Afghanistan, Cameroon and Zimbabwe have found that the primary mechanism through which PBF improved structural and process quality measures was through the availability of flexible funding at health facilities.^22 24 25^ Finally, much like in Cote d’Ivoire, PBF improved management processes in a wide range of contexts, and was in certain cases seen as a gateway to institutional reform through improving management in health centres.^7 26^

Moving forward, PBF in Cote d’Ivoire can leverage an even higher impact on quality of care through incentivising a more comprehensive definition for quality of care including provider competence, safety, continuity and population health management, as well as through measuring provider competence and patient satisfaction in a standardised manner. This can be done through regularly revising indicators to avoid undermining intrinsic motivation and disincentivising complex procedures, as well as including NCD. As it has been the case in other recent PBF programmes in Democratic Republic of the Congo and Cambodia, vignettes or direct observation can measure provider competence, and standardised patient satisfaction surveys can enable responsiveness.^27^ More importantly, PBF’s impact can be augmented by some changes at the national level. Reducing restrictions associated with PBF funds to allow facilities to purchase drugs and fuel, as well as improving the flexibility of funding arriving to health centres through block grants for essential needs of facilities, would improve PBF’s effectiveness in improving the quality of primary care. PBF is one of many reforms to improve the quality of primary care, and it should be complemented with other supply-side quality improvement interventions to ensure it reaches its maximum potential, such as improving the quality of trainings for health workers, strengthening inputs at the primary care level and increasing autonomy for health facilities. Upcoming reforms could follow the PBF pilot’s approach, which would assist in formalising a learning health system where stakeholders are incentivised to iterate and develop new solutions to improve quality of care, which would particularly improve the design of these reforms. Finally, other health system interventions with a lower administrative burden, such as global budget or capitation payments coupled with supervision to monitor and improve quality of care, could have also reached the intended impact for a specified set of system-wide goals, as can be seen from examples in China, Thailand and Kyrgyzstan, as well as countries where PBF pilots have been executed together with unconditional financing or only with supervision.^28 29^

This study has strengths and limitations. Through using an implementation science framework and assessing the pathways through which PBF implementation can reach its intended impact, it has uncovered rich contextual evidence particularly from the service delivery level, incorporating the complexity of health systems^30^ and informing changes to the programme. A limitation was the limited number of facilities sampled due to time and budget constraints; this was mitigated by ensuring variance in performance with regard to sampling. Another limitation was the deliberate focus on the health system and programme implementation context, as opposed to the political economy landscape. As PBF is a system-wide reform with significant political implications, its design and implementation are shaped by political considerations, as well as the relative interest and influence of a diverse range of stakeholders. While this context was evaluated and embedded in this assessment, it was not explicitly assessed as the main goal of this study was to demonstrate how the health systems context feeding into programme design and implementation, particularly at the health facility level, influenced programme success and implementation fidelity; this marks an important direction for future research.

## Conclusion

The Ivorian government is currently in the process of reforming its health system, with the transition to strategic purchasing under a universal health insurance scheme underway. Due to the limitations imposed by the health system context, PBF led to a successful, but partial, pattern of improvement. Despite certain shortcomings related to its design and implementation in Cote d’Ivoire, PBF has served as the entry point to orienting the country’s health system towards improving quality of care. More comprehensive health sector reforms focusing on autonomy, improved governance and integration are needed to ensure more significant improvements. These health system reforms would enable better design and implementation of PBF, ensuring maximised impact as it is scaled up and integrated within health insurance in a strategic purchasing mechanism.

With the launch of national health insurance and the national scale up of PBF, there is the potential to improve PBF’s sustainability and effectiveness in improving quality of care. In order to do so, PBF should be positioned within an integrated health financing system providing health facilities with more flexible funding and autonomy. It should only incentivise providers for a subset of indicators focusing more on cross-cutting structural and process measures, with the remainder of funds coming into facilities from health insurance reimbursements and government block grants. In addition, PBF should be complemented by more targeted quality improvement strategies aiming to improve processes and outcomes of care, such as trainings, improvements in facility infrastructure, system-wide governance and measurement reforms, and person-centeredness. Finally, it is essential for the Ivorian health system to continue learning from the strengths and shortcomings of pilots such as PBF, with a focus on enabling strategic purchasing reforms such as reduced fragmentation in financing and governance for quality of care.
